# John Hilton (1805–1878)

**DOI:** 10.1007/s00415-020-09802-7

**Published:** 2020-04-02

**Authors:** Andrzej Grzybowski, Monika Zamachowska

**Affiliations:** 1 Institute for Research in Ophthalmology, Gorczyczewskiego 2/3, 61-553 Poznan, Poland; 2 Department of the History of Medicine, Jagiellonian University Medical College, Kraków, Poland; 3 Chair of Ophthalmology, University of Warmia and Mazury, Olsztyn, Poland

**Fig. 1:**
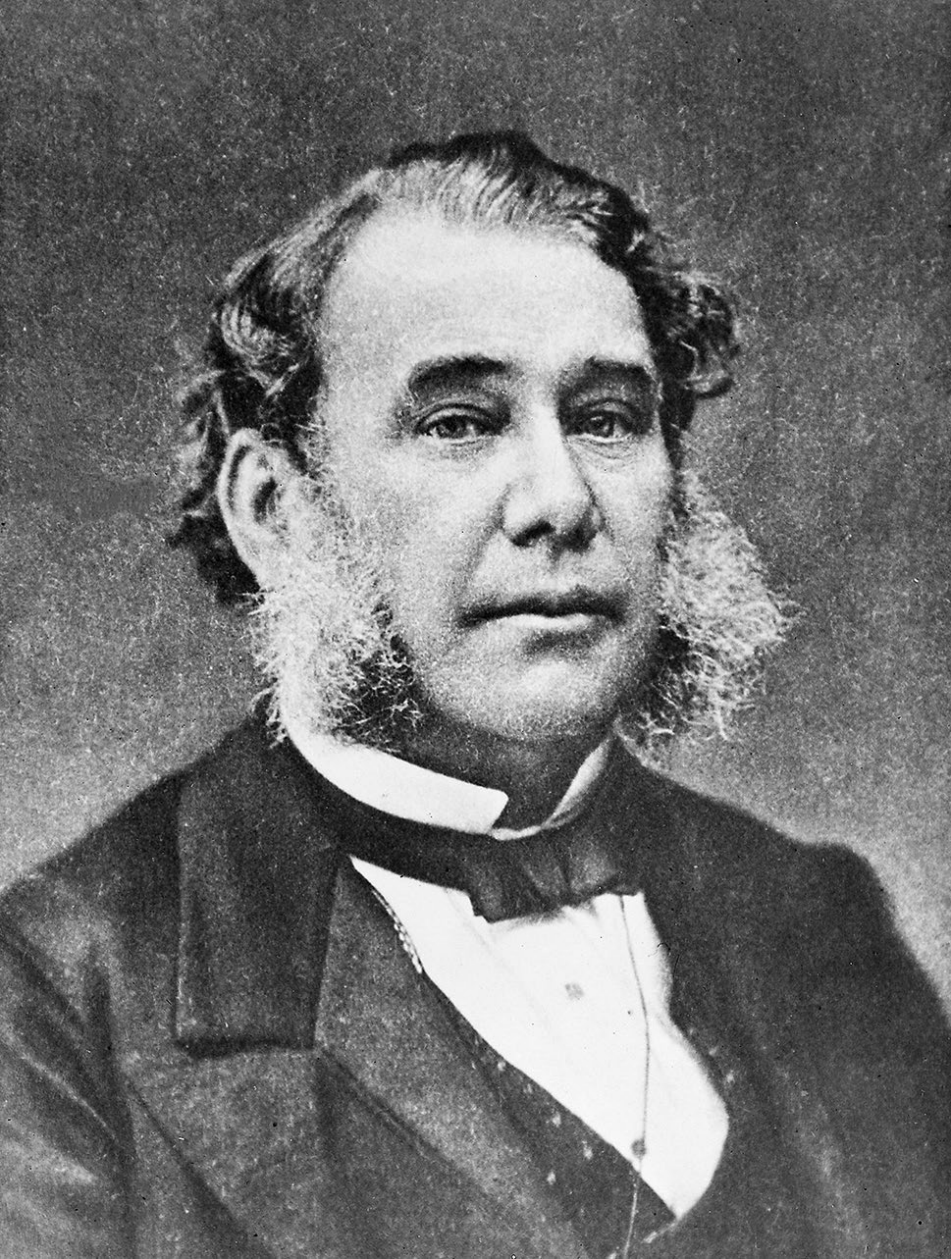
John Hilton (1805–1878) Source: https://commons.wikimedia.org/wiki/File:John_Hilton_(surgeon).jpg

John Hilton (Fig. 1) was born on 22 August 1805 in the small village of Sible Hedingham in northern Essex [1]. He was educated at King Edward VI Grammar School, Chelmsford. Later his parents financed his education in Boulogne-sur-Mer, France [1, 2]. At the beginning of 1824 John Hilton came to London, where he worked as a second demonstrator of anatomy at United Hospitals (a medical centre formed by the merger of Guy’s Hospital and St. Thomas Hospital), while still studying.

In 1825 Hilton was transferred to a new department of anatomy at Guy’s Hospital. He was a great teacher. His lectures and demonstrations gathered crowds, not only students but also experienced physicians. Thomas Addison himself attended his lectures on the nervous system, for whom a special chair was placed in the lecture hall [1]. Hilton made friends with Joseph Towne the artist. Over 13 years of collaboration (until 1838), they created a collection of anatomical wax models, which later enriched the Gordon Museum at Guy’s Hospital [1].

Hilton became a Fellow of the Royal Society in 1839. He was appointed assistant-surgeon at Guy’s Hospital in 1844 and surgeon 5 years later. Around the same time, in 1845, he was appointed a Lecturer in Anatomy at Guy’s Medical School. In 1859 he was appointed professor of human anatomy and surgery at the Royal College of Surgeons. He was also surgeon-extraordinary to Queen Victoria. From 1865 to 1867 he was vice president of the Royal College of Surgeons and in 1867 he was elected president [1]. From 1871 to 1873 he was President of the Pathological Society of London. In 1870 he retired and ended his work at Guy’s Hospital, but continued to work privately as a surgeon. In 1878, Hilton diagnosed himself with stomach cancer. The disease progressed rapidly. He died in his home in London on 14 August 1878 [1].

Hilton's anatomical observations, combined with surgical cases, led him to formulate many original, pioneering insights into anatomy, surgery and neurology.

He was the first to describe the pain resulting from compression or inflammation of nerve roots manifested by pains radiating to distant places. He described lesions in different sections of the spine that caused pain radiating to the lower limb, lower abdomen, area between the shoulder blades or chest [3, 4]. He also described various pathologies of the cranial nerves resulting in pain or dysfunction in the head and neck. As a result of his research on the damage of facial nerves and Gasserian ganglion, he described several interesting neurological tongue symptoms and unilateral mandibular tooth decay [5].

Hilton also conducted observations of diseases caused by pathological nerve compression. He claimed that if the pain in a given area is not accompanied by inflammation symptoms, it means that its cause is located in a completely different place [3].

He was the first to describe the compression of the first rib on brachial plexus causing damage to the ulnar nerve and gangrene of the fourth and fifth finger [5, 6]. Currently, the symptoms are part of the thoracic outlet syndrome (TOS).

Hilton described the circulation of cerebrospinal fluid and proved that hydrocephalus can occur after the fourth ventricle has closed [7]. He described neurological symptoms in children with hydrocephalus and the positive effects of anterior fontanelle puncture. He proved that all symptoms observed in these children are associated with the dysfunction of various centres in the brain and resolve after normalisation of cerebrospinal fluid pressure. The study suggested the possibility of future treatment of hydrocephalus through mechanical decompression [7]. He also proved that there is a connection between the subarachnoid space of the spinal cord and the brain and the free movement of fluid between these spaces [7]. He also described mental changes of depressive neurosis type in mentally fatigued patients [8]. This was one of the first such observations at the intersection of neurology and psychiatry.

As a surgeon, the Hilton was involved in treating inflammation of soft tissues and joints. He noticed that arthritis is always associated with contracture. From his observations he drew the original conclusion that inflammation covering the internal space of the joint affects all of its structures, including the nerves, which causes the motor and pain nerve endings to be constantly stimulated. The nerves innervating the joint also innervate the muscles responsible for movement in a given joint [9].

From this he concluded that the primary factor in the treatment of arthritis is the immobilization of the affected joint at the correct angle [9]. Hilton believed that the described connections between the nerves and other structures have a profound sense, as they permit maintenance of the delicate balance between mechanical forces related to muscle work and the strength of tissues both within and surrounding a joint.When this balance is upset, an overworked joint, through the activation of the nervous system, sends a message to the muscles that it needs to rest, hence the above symptoms. Based on these observations, the famous Hilton's Law was created, which says that the nerve supplying a joint also supplies both the muscles that move the joint and the skin covering the articular insertion of those muscles.

He believed that nervous system disorders may cause chronic ulcers. Observing pain reactions accompanying such ulcers, he introduced a new method of treatment consisting in cutting through the cutaneous nerves, the endings of which received pain stimuli from the region [10]. The method was widely used in medicine and led to the creation of the concept of nerve inflammation [1].

John Hilton was an outstanding anatomist and surgeon whose work also had significant implications for neurology, including the neurophysiology of pain.
